# Menstrual cycle hormonal fluctuations and their associations with athletic performance and injury susceptibility in female athletes

**DOI:** 10.3389/fspor.2026.1848550

**Published:** 2026-09-02

**Authors:** Yue Zou, Ying Gao, Ruxiang Yuan, Qinjie Yang, Tiantian Xin, Youliang Wen, Fei Xie, Xiaomin Niu, Ketao Du, Jianghua Cheng

**Affiliations:** 1 Department of Rehabilitation Medicine, South China Hospital, Medical School, Shenzhen University, Shenzhen, Guangdong, China; 2 School of Rehabilitation Medicine, Gannan Medical University, Ganzhou, Jiangxi, China; 3 Department of Rehabilitation Medicine, Dongguan Huangjiang Hospital, Dongguan, Guangdong, China

**Keywords:** anterior cruciate ligament injury, athletic performance, estrogen, injury risk, menstrual cycle, periodized training, progesterone

## Abstract

**Background:**

The menstrual cycle (MC) is characterized by periodic fluctuations in estradiol and progesterone that may influence exercise metabolism, neuromuscular function, connective-tissue properties, menstrual symptom burden, recovery, and musculoskeletal injury susceptibility in female athletes. However, inconsistent findings and substantial inter- and intraindividual variability have prevented the development of universally applicable practical guidance.

**Objective:**

This narrative review aimed to synthesize current evidence on the associations of MC-related hormonal fluctuations with athletic performance and injury susceptibility; examine the underlying physiological, biomechanical, and psychological mechanisms; and evaluate the practical implications for training, nutrition, recovery, and injury prevention.

**Main findings:**

The available evidence indicates that associations between the MC phase and objective performance outcomes are generally weak, variable, task-specific, and highly individual. Some studies have reported phase-related differences in selected measures of muscle strength, explosive performance, endurance strain, or training adaptation, whereas other studies have found no meaningful differences. Therefore, no MC phase can currently be considered universally advantageous or disadvantageous for athletic performance. Estradiol, progesterone, and relaxin provide biologically plausible pathways through which connective tissue metabolism, joint laxity, neural excitability, sensorimotor processing, and muscle-activation strategies might be modified. However, much of the supporting evidence is derived from experimental laboratory surrogate outcomes rather than prospectively recorded clinical injury data. Observational evidence linking distinct menstrual phases to anterior cruciate ligament tears, muscle strains, and other musculoskeletal injuries remains limited, heterogeneous, and unable to confirm direct causal links.

**Limitations:**

Interpretation of the evidence is constrained by small sample sizes; inaccurate or inconsistent phase classification; limited hormonal verification; inadequate control of training load; energy availability, sleep, and hormonal contraceptive use; and substantial between- and within-individual variability.

**Practical implications:**

Current evidence does not support rigid or universally applied phase-based training rules, nor arbitrary risk/performance labeling of MC phases. MC status should be considered contextual information rather than an independent basis for training prescription. Training and injury-prevention decisions should be guided primarily by repeated individual monitoring of menstrual symptoms, perceived readiness, recovery, nutritional intake, objective performance, training load, environmental stressors, and previous injury. Established neuromuscular injury-prevention and workload-management strategies should be implemented throughout the cycle rather than restricted to presumed vulnerable phases.

## 1 Introduction

The increasing participation and visibility of female athletes in competitive sports have highlighted the need to better understand female-specific physiological factors that may influence athletic performance, recovery, and susceptibility to injury. Among these factors, the menstrual cycle (MC) represents a key biological rhythm characterized by cyclic fluctuations in ovarian hormones, particularly estrogen and progesterone. These hormonal changes are not limited to reproductive function; they may also influence multiple physiological and psychological systems relevant to sports performance and athlete health, including substrate metabolism, thermoregulation, cardiorespiratory function, skeletal muscle contractility, neuromuscular control, connective tissue properties, pain perception, mood, motivation, and perceived exertion (1, 2).

From a performance perspective, MC-related hormonal fluctuations have been proposed to modify endurance responses, maximal strength, explosive power, fatigue resistance, postexercise recovery, and motor coordination (1, 2). These proposed physiological pathways are biologically plausible, but evidence for their effects on objectively measured athletic performance remains inconsistent. For example, progesterone-driven thermogenesis and ventilatory shifts may elevate thermal and perceptual strain under specific exercise and environmental conditions (2), whereas estradiol may regulate substrate metabolism, peripheral vascular function, and skeletal muscle protein turnover, as well as neural excitability (2). Crucially, these mechanistic observations do not constitute proof that athletic performance reliably rises or falls within any discrete MC phase.

From an injury-prevention perspective, epidemiological and experimental studies have reported phase-dependent differences in injury incidence, particularly for ligament injuries such as anterior cruciate ligament (ACL) tears, which occur more frequently in women (3). Changes in estrogen, progesterone, and relaxin may affect ligament laxity, collagen metabolism, joint stability, proprioception, and neuromuscular control (4, 5). These factors may be relevant to non-contact ACL injuries and other musculoskeletal injuries in female athletes (5, 6). In addition, menstrual symptoms, pain sensitivity, fatigue, and emotional changes may indirectly affect training quality, competition readiness, recovery behavior, and injury reporting (7–9).

Despite biologically plausible mechanistic links between MC-related hormonal fluctuations, athletic function, and injury susceptibility, empirical findings are highly heterogeneous across the existing literature. Several cohorts have detected phase-specific disparities in aerobic capacity, muscular strength, neuromuscular performance (2), perceived exertion, and injury prevalence, while numerous investigations report negligible or absent cycle-related alterations (1, 10). Such discrepancies are equally evident in research measuring ligament laxity and ACL injury susceptibility (5), as well as in investigations of muscle damage and postexercise recovery (11). Accordingly, although cyclical ovarian hormones can theoretically modulate performance and injury susceptibility, there is no uniform magnitude, directional trend, or clinically meaningful effect that applies to all female athletes.

Several methodological limitations may explain these inconsistent findings. First, many studies have relied on calendar-based counting or self-reported menstrual history to classify MC phases, without confirming ovulation or measuring circulating hormone concentrations (2, 12). This may lead to misclassification, especially given the substantial inter- and intraindividual variability in cycle length, ovulatory status, luteal phase duration, and hormonal amplitude. Second, sample sizes are often small, and participant characteristics vary widely in terms of training status, sport type, competitive level, menstrual symptoms, and hormonal contraceptive use (1). Third, important confounding factors, including energy availability, carbohydrate intake (13, 14), sleep, psychological stress, training load (15), recovery status (11), environmental conditions, and previous injury history, are not always adequately controlled. These limitations make it difficult to determine whether the observed changes are directly attributable to MC hormones or are influenced by other biological, behavioral, and contextual factors.

Existing reviews have addressed specific aspects of the MC in relation to exercise performance, injury susceptibility, or female athletes’ health (1, 2, 5). However, there remains a need for an integrated synthesis that links hormonal fluctuation patterns with performance outcomes (1, 2), musculoskeletal injury mechanisms (4, 5), psychological and perceptual moderators (8, 16, 17), methodological controversies, and practical training implications (12, 18, 19). Such an approach is important because MC effects are unlikely to operate through a single pathway. Instead, they may emerge from interactions among endocrine, metabolic, neuromuscular, biomechanical, connective tissue, and psychosocial factors.

Therefore, this review aims to synthesize current evidence regarding the associations between MC-related hormonal fluctuations, athletic performance, and musculoskeletal injury susceptibility in female athletes.

Specifically, this review seeks to

1. Summarize the characteristic hormonal fluctuation patterns across MC phases;
2. Evaluate the potential effects of these fluctuations on aerobic endurance (20), maximal strength and explosive power (21), and neuromuscular control and motor coordination (22, 23);
3. Examine their associations with ACL injury (3, 5), muscle strain, stress fracture, and generalized joint laxity (24, 25), alongside other musculoskeletal injuries;
4. Discuss the moderating roles of menstrual-related psychological symptoms, subjective perceived exertion, pain perception (8, 17), and postexercise recovery status (11, 26);
5. Identify key methodological challenges that contribute to inconsistent empirical findings (1, 2, 12); and
6. Discuss practical implications for individualized MC and symptom monitoring (12, 27), general sports nutrition and recovery support (28–31), and directions for future research, while distinguishing established guidance from unverified phase-specific interventions.

Several recent systematic reviews, narrative reviews, and position statements have examined the MC phase in relation to exercise performance, neuromuscular function, injury susceptibility, or nutritional requirements in female athletes. However, these prior works typically discuss each domain in isolation, which creates two key limitations: first, it becomes difficult to clearly distinguish direct clinical outcome evidence from purely mechanistic or surrogate biomarker evidence; second, heterogeneous scattered findings cannot be readily translated into balanced, proportionate practical guidance for practitioners. Notably, a common interpretive flaw in the existing literature is that associations among sex hormone concentrations, ligament laxity, and neuromuscular metrics are frequently overinterpreted as definitive proof of phase-specific injury susceptibility, even though the complete causal chain linking hormonal fluctuations to actual clinical injuries remains incompletely verified. Against this backdrop, the novelty of the present narrative review lies in its unified, integrated analytical framework, rather than merely cataloging scattered phase-related physiological differences as prior summaries do. This work systematically integrates core dimensions, including hormonal physiology, athletic performance, injury susceptibility, psychological-perceptual moderators, methodological limitations in existing research, and actionable applied training strategies. A central distinguishing feature of this synthesis is that it explicitly stratifies evidence by consistency and causal certainty, strictly separating observed real-world performance/injury endpoints from theoretical mechanistic pathways and laboratory surrogate markers, to avoid overly deterministic claims when empirical data remain insufficient. By simultaneously highlighting the biologically plausible relevance of MC-related hormonal fluctuations alongside widespread cross-study uncertainty and interindividual variability, this review delivers a nuanced, balanced perspective tailored for athletes, coaches, clinicians, and sports science researchers. Most critically, the cumulative body of available evidence fails to justify rigid, one-size-fits-all phase-based training protocols for all female athletes. A more proportionate current approach is to treat MC information as one component of individualized monitoring and to make flexible training decisions based on each athlete's reproducible symptom profile, readiness, training load, recovery status, and objective performance responses (1, 12, 18).

## 2 Methods

This narrative review draws on targeted searches of PubMed, Web of Science, and Scopus to assemble relevant published evidence, with searches spanning from database inception to July 2026 and no language restrictions imposed. We constructed search strategies using combined keywords pertaining to MCs and female athletes, including “menstrual cycle,” “menstrual phase,” “estrogen,” “estradiol,” “progesterone,” “female athlete,” “exercise performance,” “muscle strength,” “cardiorespiratory endurance,” “neuromuscular control,” “injury susceptibility,” “ACL,” and “sports injury.” Additional records were identified through manual screening of the reference lists of included original papers and related reviews. Abstract and full-text screening were performed independently by two authors, with disagreements settled through group discussion. Table 1 provides a purposive, non-exhaustive selection of original studies examining performance, recovery, or injury-related outcomes. Studies were selected to illustrate variation in participant characteristics, outcome domains, hormonal contraceptive status, and MC assessment methods, ranging from calendar-based estimates and bleeding records to urinary luteinizing hormone (LH) testing and biochemical hormone verification. The table was not used as the sole basis for the conclusions of the review.

**Table 1:** Representative studies examining MC-related athletic performance, recovery, and injury outcomes.

Study	Population	Phase confirmation	Hormonal contraception	Outcomes and main findings
Rael et al. (20)	21 endurance-trained women	Calendar, urinary LH, and serum E2/P4	Excluded	Most cardiorespiratory and perceptual outcomes remained unchanged; selected heart rate and ventilatory responses differed across phases
Thompson et al. (45)	30 active women recruited; 28 included in the final analysis (10 naturally menstruating, 10 high-androgenicity oral-contraceptive users, and eight low-androgenicity oral-contraceptive users)	Calendar, urinary LH, and serum E2/P4 for naturally menstruating participants; pill schedule for users	Naturally menstruating and oral-contraceptive groups were analyzed separately	Most strength and perceptual outcomes were stable, although selected explosive outcomes differed across cycle conditions
Romero-Moraleda et al. (49)	15 elite, naturally menstruating female football players	Calendar, urinary LH, salivary hormones, and temperature	Excluded	Jump performance, concentric velocity, and perceived exertion remained broadly stable across phases
Silva et al. (26)	20 amateur female football players	Calendar-based estimation	Excluded	RSI and salivary IL-6 differed between calendar-estimated early follicular and midluteal phases, whereas DOMS did not show a significant phase-related difference. Interpretation is limited by the absence of ovulation or hormone verification
Ferrer et al. (61)	33 elite female football players were followed for four seasons	Bleeding records; bleeding vs. non-bleeding days	Excluded	Injury incidence did not differ significantly between bleeding and non-bleeding days. Injury burden was higher during bleeding, but only 11 injuries occurred during bleeding; this finding should therefore be considered exploratory

DOMS, delayed-onset muscle soreness; E2, estradiol; IL-6, interleukin-6; LH, luteinizing hormone; P4, progesterone; RSI, reactive strength index. Phase-confirmation methods vary substantially across studies. Calendar-based estimates, bleeding records, symptom records, and basal body temperature measurements cannot independently confirm ovulation or the underlying hormonal milieu. Laboratory or biomechanical injury-risk surrogates should not be interpreted as equivalent to prospectively recorded clinical injuries. The studies shown are representative rather than exhaustive.

Studies were considered for inclusion if they examined MC-related hormonal fluctuations in relation to athletic performance, exercise responses, recovery, psychological or perceptual outcomes, or susceptibility to musculoskeletal injury in physically active women or female athletes. Original research articles, systematic reviews, meta-analyses, narrative reviews, consensus statements, and position stands were considered when relevant to the scope of this review. Studies focusing exclusively on pregnancy, menopause, or clinical populations that were not relevant to exercise or sports performance were excluded. We prioritized peer-reviewed papers that clearly reported MC assessment protocols, athlete demographics, and outcomes related to athletic performance or musculoskeletal injury.

Because the purpose of this article was to provide a broad, interdisciplinary interpretation of the literature rather than to answer a narrowly defined clinical question, the evidence was synthesized narratively. The findings were organized into the following themes: hormonal patterns and MC phase identification; metabolic and cardiorespiratory performance; muscle strength and explosive power; neuromuscular control; musculoskeletal injury susceptibility; psychological and perceptual factors; and cycle-informed training, nutritional, and recovery strategies. No formal meta-analysis or risk-of-bias assessment was conducted. Therefore, this review should be interpreted as a narrative synthesis rather than a systematic review conducted in accordance with the PRISMA framework.

To reduce causal overinterpretation, the evidence was evaluated based on the type and strength of inference permitted by the study design and outcome. Findings reproduced across evidence syntheses or multiple appropriately designed studies were described as relatively consistent findings. Relationships derived mainly from cross-sectional, retrospective, or other observational studies were described as limited or heterogeneous observational associations. Pathways supported primarily by *in vitro*, animal, tissue-level, neurophysiological, or biomechanical studies were treated as biologically plausible mechanisms rather than established causes of performance changes or injury. Laboratory measures, including ligament laxity, muscle activation, cortical excitability, postural control, dynamic knee valgus, and anterior tibial translation, were classified as mechanistic or surrogate outcomes and were not treated as equivalent to prospectively recorded clinical injuries. Relationships not directly supported by prospective clinical outcomes were explicitly described as hypotheses requiring further validation. Because this article is a narrative review, these categories were used to guide the interpretation and wording of the evidence rather than as a formal evidence-grading system.

## 3 Hormonal fluctuation patterns and phases of the menstrual cycle

### 3.1 Secretion patterns and physiological functions of major sex hormones

The concentrations of key reproductive hormones, including estradiol, progesterone, LH, and follicle-stimulating hormone (FSH), fluctuate dynamically across the MC. As the primary circulating estrogen in reproductive-age women, estradiol exhibits a prominent peak in the late follicular phase before ovulation and a smaller secondary elevation in the midluteal phase. Experimental physiological evidence indicates that estradiol may modulate vascular function, substrate metabolism, muscle contractility, and neural excitability (2, 32, 33). Importantly, such mechanistic observations do not confirm that elevated estradiol levels directly translate to superior athletic performance. Progesterone is synthesized mainly by the corpus luteum postovulation and peaks in the midluteal phase. It drives the postovulatory rise in basal body temperature and may alter ventilatory control and central neural inhibitory signaling (2, 34). The effects of progesterone on connective tissue and neuromuscular function are still poorly characterized; these associations are biologically plausible pathways rather than definitive predictors of exercise capacity or musculoskeletal injury susceptibility.

**Figure 1:**
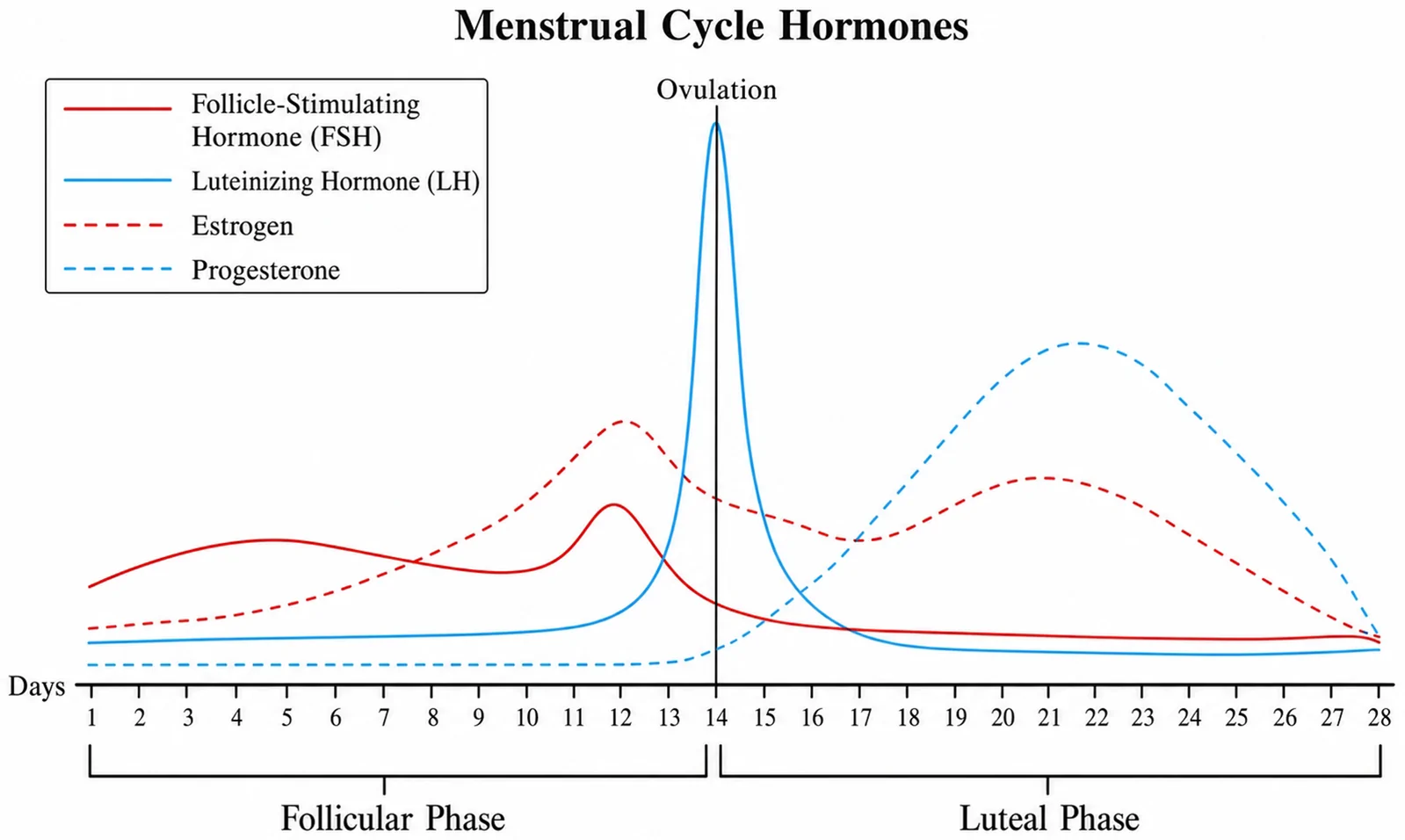
Diagram of hormonal fluctuations across the female menstrual cycle.

LH and FSH, secreted by the anterior pituitary, regulate follicular development and ovulation through feedback interactions with estradiol and progesterone. FSH increases during the early follicular phase and supports follicular development and estradiol production. A transient LH surge occurs immediately before ovulation and triggers follicular rupture and oocyte release (2). Beyond their reproductive functions, ovarian hormones may influence cardiovascular regulation, substrate metabolism, connective-tissue turnover, neuromuscular function, and behavioral responses. For example, estradiol may modulate vascular reactivity partly through its effects on vascular potassium channels, whereas progesterone and its neuroactive metabolites may influence neural excitability and inhibitory signaling (32, 34). MC-related hormonal fluctuations have also been associated with changes in perceived stress, motivation, and exercise-related perceptions (35, 36). However, these associations are heterogeneous and do not demonstrate that circulating hormone concentrations directly determine athletic performance or injury susceptibility. Overall, the coordinated fluctuations in estradiol, progesterone, LH, and FSH provide a physiological framework for investigating possible cycle-related changes, but their functional consequences must be evaluated using direct performance and clinical injury outcomes. The detailed temporal pattern of ovarian and pituitary hormones across a standard cycle is visualized in Figure 1. (Note that this schematic represents a generalized 28-day MC profile for illustrative purposes only and is not based on individual empirical data.) These phase-specific hormonal signatures establish a physiological framework to explore potential cycle-dependent variability in physiological exercise responses and injury outcomes (Table 2). Importantly, these hormonal patterns alone cannot confirm phase-linked alterations in athletic performance or clinical injury susceptibility. Higher estradiol concentrations during the preovulatory period have been proposed to influence substrate utilization and neural excitability, whereas elevated progesterone during the luteal phase contributes to postovulatory thermogenesis and may influence selected ventilatory or perceptual responses. However, these physiological observations have not been shown to produce a consistent late-follicular performance advantage or a consistent luteal-phase performance disadvantage. Associations with joint laxity remain inconsistent and should not be interpreted as evidence of increased susceptibility to clinical injury.

**Table 2:** Summary of hormonal profiles and potential performance-related effects across the menstrual cycle.

MC phase	Typical hormonal profile	Potential physiological mechanisms	Potential performance effects	Overall interpretation of evidence
Early follicular phase, overlapping with menstruation	Low E2 and P4; FSH begins to rise	Possible symptom burden associated with menstrual bleeding, including pain or fatigue in some individuals	Performance generally maintained; possible symptom-related impairment	Mixed; mostly trivial effects
Late follicular	Rising E2; low P4	Proposed changes in neural excitability, vascular function, substrate use, and muscle contractility	Small differences in selected strength or high-intensity outcomes have been reported; most outcomes remain unchanged	Mixed; small effects
Ovulatory	LH surge; peak or declining E2; low-to-rising P4	Proposed differences in selected neural, connective-tissue, or joint-laxity measures	Selected studies have reported differences in strength or coordination measures; objective performance is often unchanged	Limited and inconsistent
Early luteal	Rising P4; moderate E2	Increasing thermogenesis and altered ventilatory regulation	Performance generally maintained	Limited evidence
Midluteal	High P4; secondary E2 rise	Higher postovulatory body temperature; proposed differences in ventilation, substrate use, or neurophysiological measures	Possible differences in perceived thermal or exercise strain in some individuals; objective performance is often unchanged	Mixed; task-dependent
Late luteal/premenstrual	Falling E2 and P4; possible symptom onset	Declining E2 and P4; possible mood, sleep, or pain-related symptoms in susceptible individuals	Possible increases in perceived fatigue or soreness and lower self-reported readiness among symptom-affected individuals	Individual and symptom-dependent

E2, estradiol; P4, progesterone; FSH, follicle-stimulating hormone; LH, luteinizing hormone. Hormonal profiles represent typical ovulatory cycles and may vary substantially within and between individuals. Reported performance effects are generally small and inconsistent and should not be interpreted as supporting universal phase-based training prescriptions. Menstruation overlaps with the early follicular phase of the ovarian cycle.

**Table 3:** Symptom-informed considerations across menstrual cycle phases.

Phase or context	Main considerations	Practical recommendations	Interpretive basis and key limitations
Early follicular/menstruation	Some athletes experience pain, fatigue, heavy bleeding, or gastrointestinal symptoms, whereas others remain unaffected	Maintain usual training when symptoms are mild. Consider temporary symptom-guided adjustments. Assess iron status when clinically indicated; do not prescribe iron routinely without biochemical assessment	Individual symptoms are clinically relevant, but evidence does not support routine phase-based load reduction
Late follicular/ovulatory	Small differences in selected performance or biomechanical outcomes have been reported, but findings are inconsistent	Training may proceed as planned. Do not classify this phase as universally optimal for performance or high risk for injury	Most evidence concerns small performance effects or laboratory surrogates rather than clinical injury
Midluteal	Postovulatory increases in body temperature may contribute to greater thermal discomfort in some athletes, particularly in hot environments	Maintain usual training and nutrition unless reproducible symptoms are present. Consider individualized hydration, cooling, or recovery adjustments when heat intolerance or impaired recovery is repeatedly observed	Thermoregulatory changes are biologically plausible, but direct evidence supporting routine luteal-phase nutritional or recovery modification is limited
Late luteal/premenstrual	Fatigue, sleep disturbance, mood changes, soreness, or higher perceived exertion may occur in susceptible athletes	Maintain usual training and nutrition when symptoms are mild. When symptoms are reproducible and functionally relevant, consider temporary adjustments to training volume, session density, recovery, or food intake according to individual needs	Symptom effects are heterogeneous. Adequate energy and carbohydrate availability are general sports nutrition priorities, not interventions that have been directly validated for the late luteal phase
All phases and hormonal contraceptive users	Responses vary among individuals, cycles, sports, and hormonal-contraceptive formulations	Apply established training and sports nutrition principles throughout the cycle. Monitor symptoms, readiness, recovery, dietary intake, training load, and performance over time. Do not apply natural-cycle phase rules directly to hormonal-contraceptive users	Individual longitudinal monitoring is better supported than universal phase-based training, nutrition, supplementation, or recovery prescriptions

FSH and LH peak around ovulation; estrogen increases during the late follicular phase and has a smaller increase in the midluteal phase; progesterone is low in the follicular phase and increases during the midluteal phase. This figure is a generalized conceptual illustration rather than a plot of raw individual participant data. Figure 1 was drawn *de novo* by the authors using Adobe Photoshop 2024 (version 25.0) as a generalized conceptual illustration of established MC endocrinology (2); no third-party image or figure was reproduced or adapted, and no additional copyright permission is required.

### 3.2 Hormone-based menstrual cycle phase classification

The MC can be described using ovarian- and uterine-cycle frameworks. From an ovarian-cycle perspective, the principal stages are the follicular phase, ovulation, and the luteal phase. Menstruation is a uterine-cycle event that usually occurs during the early follicular phase; it therefore overlaps with that phase rather than constituting a separate ovarian-cycle phase. During the follicular phase, estradiol concentrations generally rise as the dominant follicle develops, whereas progesterone remains relatively low. Estradiol typically reaches a preovulatory peak, followed by an LH surge and ovulation. After ovulation, progesterone rises as the corpus luteum develops, and both progesterone and estradiol are usually elevated during the midluteal phase (2, 12). These profiles represent typical ovulatory cycles and should not be regarded as fixed patterns for all individuals or cycles.

In this review, menstruation is treated as a bleeding event that generally overlaps with the early follicular phase rather than as a separate ovarian-cycle phase. When original studies used “menstrual phase” as an operational testing window, their terminology is reported as author-defined, without implying that menstruation constitutes an independent ovarian-cycle phase.

Accurate identification of MC phases is essential when investigating associations between hormonal fluctuations, athletic performance, and injury-related outcomes. Calendar-based estimation alone is insufficient because cycle length, ovulation timing, luteal-phase duration, and hormonal amplitude vary both between and within individuals. Regular bleeding patterns do not by themselves confirm ovulation or adequate luteal progesterone exposure. Anovulatory cycles or inadequate luteal progesterone exposure can occur despite apparently regular bleeding, resulting in hormonal profiles that may differ from those predicted by calendar counting (37). Wearable-derived physiological measures, such as the sleeping heart-rate nadir, have recently been investigated as potential tools for phase classification and ovulation detection under free-living conditions (38). However, these approaches remain complementary to, rather than substitutes for, biochemical verification.

Biochemical assessment provides the most direct method of characterizing the hormonal milieu. Serum measurements of estradiol and progesterone are commonly used, although larger-volume capillary blood sampling may offer a more practical alternative for field-based studies (39). Urinary progesterone metabolites, such as pregnanediol glucuronide, can provide non-invasive information about luteal progesterone activity and ovulatory status (40). Urinary LH testing can improve the estimation of ovulation timing, but an LH surge alone does not confirm successful ovulation or adequate luteal progesterone exposure. Therefore, combining prospective cycle tracking, urinary LH testing, and measurements of estradiol and progesterone provides a more rigorous approach to phase classification (12, 39, 40).

Accurate MC phase classification is important when evaluating whether exercise- and injury-related outcomes vary with the hormonal milieu. Selected studies have reported between-phase differences in outcomes such as VO_2_max, eccentric knee-extension torque, pain sensitivity, and autonomic measures (17, 37, 41, 42). However, these findings were obtained from different populations, protocols, outcomes, and phase-confirmation methods and have not been consistently reproduced. They should therefore be interpreted as examples of possible hormone-associated variation rather than evidence that these outcomes reliably change across the MC. More accurate phase classification may reduce measurement error and help determine whether reported differences reflect hormonal variation, other contextual factors, or normal within-person variability.

From a practical perspective, MC phase identification can be organized into a three-level methodological hierarchy. The first level consists of self-reported bleeding dates, calendar tracking, and symptom records. Although feasible for routine monitoring, this approach cannot confirm ovulation or the underlying hormonal milieu. The second level combines self-reported cycle information and symptom monitoring with urinary LH testing, which improves the estimation of ovulation timing but does not independently confirm successful ovulation or adequate luteal progesterone exposure. The third and most rigorous level involves hormonal verification using estradiol and progesterone measurements in serum or capillary blood, preferably combined with urinary LH testing (12, 39, 43). Self-report may be appropriate for routine symptom monitoring, whereas mechanistic studies and intervention trials should use ovulation detection and biochemical hormone verification whenever feasible.

The characteristic hormonal profiles of the main MC phases and their potential associations with exercise performance are summarized in Table 2. Importantly, these patterns represent typical group-level profiles rather than fixed individual trajectories, and the magnitude and direction of performance changes vary substantially among individuals and studies.

## 4 Potential mechanisms and evidence regarding associations between menstrual cycle hormonal fluctuations and athletic performance

### 4.1 Potential associations with metabolism and cardiorespiratory endurance

MC-related variation in estradiol and progesterone has been proposed to influence substrate metabolism, thermoregulation, ventilation, and perceived exercise strain. Experimental physiological studies suggest that estradiol may affect lipid and carbohydrate metabolism under some exercise conditions, whereas progesterone contributes to the postovulatory increase in body temperature and may influence ventilatory regulation (2, 33). However, these mechanisms are context-dependent and may vary with exercise duration and intensity, nutritional status, training status, and environmental temperature. They do not demonstrate that elevated estradiol consistently improves endurance performance or that elevated progesterone consistently impairs it.

Studies of objectively measured aerobic and endurance outcomes have produced mixed findings. Selected studies have reported phase-related differences in VO_2_max, time-trial performance, heart rate, ventilation, or perceived strain, whereas many others have found little or no meaningful variation across hormonally distinct phases (1, 2, 10, 20). Where differences have been observed, their magnitude has generally been small, and their direction has not been consistent across protocols or populations. It is therefore not appropriate to characterize the late follicular phase as a universally advantageous endurance window or the luteal phase as a universally disadvantageous period.

In endurance-trained eumenorrheic women, Rael et al. evaluated cardiorespiratory responses to high-intensity interval exercise across the early follicular, late follicular, and midluteal phases (20). Selected ventilatory and heart rate responses differed across phases, whereas oxygen uptake, carbon dioxide production, the respiratory exchange ratio, breathing frequency, energy expenditure, rating of perceived exertion, and perceived readiness remained largely unchanged. These findings indicate that small phase-related differences may occur in selected monitoring variables, but they do not establish a meaningful phase effect on overall exercise capacity. Heart rate data should therefore be interpreted alongside external workload, perceived exertion, environmental conditions, and individual longitudinal responses rather than automatically adjusted according to the MC phase.

Comparisons among premenopausal women, hormonal contraceptive users, and postmenopausal women may provide insight into broad associations between reproductive-hormone environments and cardiorespiratory physiology (44). However, the substantial endocrine differences associated with menopause are not equivalent to normal within-cycle fluctuations and cannot be used to infer a late-follicular performance advantage. Similarly, findings in users of specific oral-contraceptive formulations should not be generalized to naturally menstruating athletes or to all contraceptive types. Overall, current evidence indicates that any association between the MC phase and cardiorespiratory endurance is likely to be weak, task-dependent, and highly individual.

### 4.2 Potential associations with muscle strength and explosive performance

Evidence regarding MC-related changes in muscle strength and explosive performance remains inconsistent. One study reported greater isokinetic knee flexion torque at a high angular velocity (240°·s⁻^1^) and longer flight time in bilateral hopping and counter-movement jumps during the midluteal phase compared to the late follicular phase, whereas isometric strength and low-velocity torque remained unchanged (45). Other studies, in contrast, have reported strength differences in other cycle phases or no meaningful phase-related differences (46–48). Studies on elite athletes have likewise reported relatively stable jump performance, concentric velocity, and perceived exertion across MC phases (49). These findings are not necessarily directly contradictory because the studies assessed different muscle groups, contraction modes, angular velocities, exercise tasks, and participant populations.

At the evidence-synthesis level, the MC phase appears to have, at most, a small and inconsistent influence on acute maximal strength and explosive performance (1, 2, 10, 50). Some analyses have reported marginally elevated values for several isometric and dynamic strength metrics during late-follicular testing sessions, as defined by calendar tracking or hormonal measurements (50). Nevertheless, the magnitude of these detected differences is modest, the overall certainty of supporting evidence is low, and current datasets cannot confirm that the concurrent estradiol–progesterone hormonal profile is the causal driver of such strength variations. In addition, other studies have reported higher values during the midluteal phase (45) or no phase-related differences in peak torque, fatigue resistance, or sport-specific neuromuscular performance (47–49). The late follicular phase therefore cannot currently be regarded as a universally optimal phase for strength or power performance.

Estradiol has been proposed to influence skeletal muscle contractile function, protein turnover, recovery, and neural excitability (2, 51). These mechanisms provide hypotheses for investigating phase-related variation, but they do not establish that higher estradiol exposure produces superior strength or power performance. Progesterone and its neuroactive metabolites may also modulate central nervous system excitability and could theoretically modify voluntary muscle activation or rapid force production (34, 52). Nevertheless, estradiol and progesterone are simultaneously elevated during the midluteal phase, and their physiological effects cannot be interpreted independently. Moreover, circulating hormone concentrations do not directly determine muscle force production or motor unit behavior. A recent preprint reported statistically significant but small associations between MC-related hormone concentrations and selected motor-unit discharge properties (53). These preliminary findings require peer-reviewed replication and should not be interpreted as evidence of meaningful changes in whole-muscle performance.

The evidence concerning explosive performance is similarly inconsistent. Some studies have reported phase-related differences in isokinetic strength or repeated-sprint responses (45, 54), whereas others have found no meaningful changes in jump height, concentric velocity, peak torque, or fatigue resistance across the cycle (48, 49). Greater fatigue, muscle soreness, or muscle-damage responses have occasionally been observed during the author-defined premenstrual phase (54), but systematic evidence regarding exercise-induced muscle damage remains inconclusive (11). These findings indicate that muscle damage, neuromuscular fatigue, and acute explosive performance should be considered as related but distinct outcomes.

Acute strength performance should also be distinguished from long-term resistance-training adaptation. A small number of preliminary studies have reported greater improvements in selected strength or muscle mass outcomes when more resistance-training sessions were scheduled during the follicular phase than during the luteal phase (51). However, the interpretation of these findings is limited by small sample sizes, imprecise phase classification, differences in training frequency and volume, and insufficient replication. Current higher-level evidence does not establish that follicular-phase-based resistance training produces consistently greater strength or hypertrophic adaptations than conventional or luteal-phase-based training (10). Therefore, the evidence is currently insufficient to recommend systematically concentrating high-load resistance training in a particular MC phase.

Hormonal contraceptive use introduces additional complexity because exogenous hormones suppress or modify endogenous ovarian-hormone fluctuations. Available evidence suggests that monophasic oral contraceptives do not substantially alter acute muscle strength in most users, although they may influence some markers of recovery after exercise-induced muscle damage (55). Differences among contraceptive formulations, including androgenicity, dose, and withdrawal schedules, may also contribute to variations in muscle-performance findings (45, 55). Studies should therefore analyze naturally menstruating athletes and hormonal contraceptive users separately rather than treating them as a homogeneous population.

Overall, current evidence does not demonstrate a consistent or practically meaningful advantage of either the late follicular or midluteal phase for muscle strength and explosive performance (1, 10, 48, 50). Small phase-related differences may occur in selected outcomes or individuals, but their direction and magnitude depend on the measurement method, exercise task, training status, hormonal profile, and accuracy of cycle-phase confirmation. Accordingly, individualized monitoring of symptoms, recovery, and actual performance responses is more appropriate than universal phase-based strength-training prescriptions.

### 4.3 Potential associations with neuromuscular control and coordination

Ovarian hormone fluctuations have been investigated in relation to selected neurophysiological and neuromuscular outcomes, but evidence of consistent phase-related changes in whole-body motor control remains limited. Estradiol has been proposed to modulate spinal reflex excitability, muscle spindle sensitivity, and cortical function. However, elevated estradiol cannot be assumed to improve or impair neuromuscular control because task demands, fatigue, training status, and individual neural adaptation may modify the observed response.

Progesterone can be metabolized to the neuroactive steroid allopregnanolone, which acts as a positive allosteric modulator of GABA(__A_) receptors (34, 56). This established molecular pathway does not necessarily imply a phase-specific change in sport-related coordination, because central neurosteroid concentrations, receptor sensitivity, and task-level motor responses are not determined solely by circulating progesterone. Some transcranial magnetic stimulation studies have reported greater intracortical inhibition during phases characterized by relatively high progesterone concentrations, particularly the luteal phase (52, 57). However, these findings are not entirely consistent, and this mechanism should therefore be interpreted cautiously (52).

Several factors limit the direct interpretation of this pathway. First, circulating progesterone concentrations do not directly reflect central allopregnanolone concentrations or GABA(__A_)-receptor sensitivity, both of which may vary among individuals (34, 56). Second, estradiol and progesterone are simultaneously elevated during the midluteal phase and may exert partly different effects on neural excitability. It is therefore difficult to attribute phase-related changes in cortical excitability exclusively to progesterone. Third, transcranial magnetic stimulation measures, such as short-interval intracortical inhibition and the cortical silent period, are indirect neurophysiological markers. They do not directly quantify brain GABA concentrations and should not be considered equivalent to changes in whole-body coordination or sport-specific performance (52).

The translation of changes in cortical excitability into sensorimotor integration and athletic coordination also remains uncertain. Sensorimotor performance depends on the integration of visual, vestibular, and proprioceptive information, as well as on attention, task familiarity, fatigue, pain, and training status. Studies examining postural control and neuromuscular function across the MC have reported mixed findings (22, 23, 58). Therefore, although complex motor tasks may theoretically be sensitive to hormonal fluctuations, current evidence does not establish the follicular phase as a universally superior period for complex skill acquisition or demonstrate that the luteal phase consistently impairs coordination.

Some studies have examined neuromuscular responses across different MC phases. For example, repeated-sprint exercise in eumenorrheic female handball players was associated with greater reductions in neuromuscular efficiency and maximal voluntary contraction during the premenstrual phase than during other assessed phases (54). However, this finding was derived from a specific exercise protocol and athlete population and should not be generalized to all forms of neuromuscular performance or all female athletes. It nevertheless suggests that MC-related symptoms and recovery responses may influence selected neuromuscular outcomes in some individuals.

Overall, MC-related hormonal fluctuations may modulate neural excitability, sensorimotor processing, and selected aspects of neuromuscular function. Estradiol may influence spinal and cortical excitability, whereas progesterone and its neuroactive metabolites may modify GABAergic inhibition (34, 52, 56, 57). Nevertheless, direct evidence linking these neurophysiological mechanisms to meaningful changes in coordination or sport-specific performance remains limited and inconsistent. These mechanisms should therefore be regarded as plausible explanatory pathways rather than established causes of phase-specific performance changes.

Accordingly, neither elevated estradiol during the late follicular phase nor elevated progesterone during the luteal phase should be used to predict superior or impaired motor control in an individual athlete.

## 5 Evidence regarding menstrual cycle hormonal fluctuations and sports injury susceptibility

Sports injuries are multifactorial events that cannot be explained by MC-related hormonal effects alone. Hormonal fluctuations may alter ligament characteristics, joint laxity, neuromuscular function, symptom severity, and recovery capacity; however, injury susceptibility is additionally shaped by anatomical structure, previous injury, movement mechanics, training and competition load, fatigue, playing surface, and other environmental and psychological factors (5, 59, 60). Moreover, many MC studies have assessed laboratory-based injury-risk surrogates, such as knee laxity, muscle activation, or dynamic knee valgus, rather than prospectively recorded clinical injuries (5). Current studies do not identify a consistently high-risk MC phase across all injuries, sports, and athlete populations (5, 61). Therefore, the following hormonal and biomechanical pathways should be interpreted as potential contributing mechanisms rather than evidence that a particular MC phase directly causes sports injury.

### 5.1 Proposed biomechanical and hormonal pathways relevant to anterior cruciate ligament injury susceptibility

Female athletes have a higher incidence of ACL injury than male athletes in several comparable sports, although the magnitude of this difference varies according to sport, exposure, competition level, and study design. Some observational studies have reported a greater proportion of non-contact ACL injuries during preovulatory or peri-ovulatory windows. However, phase definitions and verification methods vary substantially, and no consistently high-risk MC phase has been established (60, 62, 63). ACL injury is multifactorial and may be influenced by anatomical morphology, previous injury, movement strategy, neuromuscular control, fatigue, training and competition exposure, playing surface, and environmental conditions (59, 60, 63, 64). MC-related hormonal fluctuations should therefore be considered possible modifiers within a broader risk framework rather than independent causes of injury.

At the mechanistic level, estrogen receptors have been identified in ligament fibroblasts, and *in vitro* and preclinical studies suggest that estradiol may influence collagen synthesis, matrix-remodeling activity, and selected mechanical properties of ligament tissue (4, 65). These findings provide biological plausibility for a hormone-ligament interaction. However, cellular changes observed under experimental conditions cannot be assumed to produce clinically meaningful reductions in ACL strength or stiffness *in vivo*. Moreover, a recent systematic review and meta-analysis found no overall clinically meaningful effect of the MC phase on knee laxity or anterior tibial translation, despite evidence of hormonal effects in selected cellular and preclinical models (4). Therefore, elevated estradiol during the late follicular or peri-ovulatory period should not be interpreted as directly weakening the ACL or increasing injury susceptibility.

Alterations in neuromuscular control constitute a second key pathway through which hormonal variations may modify ACL injury susceptibility. Studies have examined whether MC-related hormonal fluctuations are associated with changes in quadriceps–hamstring activation, landing mechanics, dynamic knee valgus, proprioception, and postural control. However, findings are inconsistent, and the available evidence does not demonstrate a reproducible phase-specific pattern of quadriceps dominance or reduced hamstring coactivation (5, 66). A systematic review of jump-landing studies identified some sex-related differences in lower-limb muscle activation, but these findings cannot be attributed specifically to MC hormones (67). Moreover, electromyographic and biomechanical measures are surrogate outcomes and do not directly establish susceptibility to clinical ACL injury. Established neuromuscular training programs should therefore be implemented throughout the training year rather than restricted to a presumed hormonally vulnerable phase.

A third proposed pathway involves progesterone and relaxin. One study reported differences in anterior knee laxity, stiffness, genu recurvatum, and generalized joint laxity between the late follicular and ovulatory phases (68), but these findings have not been reproduced consistently. The hypothesis that transient changes in passive tissue compliance could interact with dynamic muscular stabilization during cutting or landing is biomechanically plausible; however, it has not been established as a cycle-dependent cause of ACL injury (5, 66). Relaxin has also been investigated in relation to extra-articular connective tissues, but evidence that normal within-cycle variation in circulating relaxin meaningfully alters knee biomechanics or clinical injury susceptibility remains insufficient (3, 4).

Overall, tissue-level studies support the biological plausibility of hormone-sensitive ligament metabolism, whereas human studies on knee laxity, muscle activation, and landing biomechanics remain heterogeneous (4, 5, 69). These laboratory outcomes should be classified as mechanistic or surrogate measures rather than clinical injuries. Current evidence does not demonstrate that a hormone-associated change in any single surrogate outcome produces a clinically meaningful increase in ACL injury incidence during a specific MC phase.

**Figure 2:**
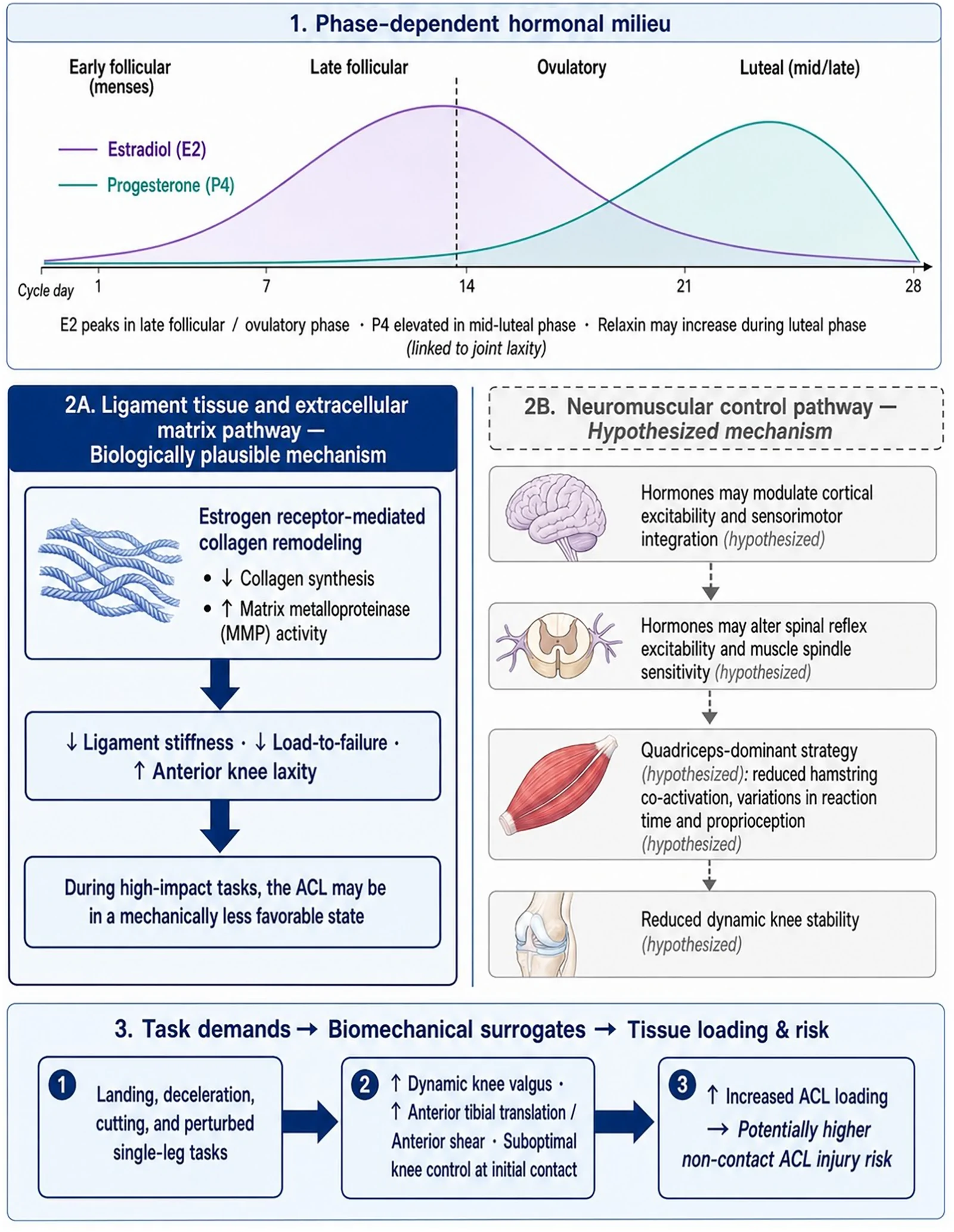
Potential mechanisms linking menstrual cycle-related hormonal variation to susceptibility to non-contact ACL injury.

Figure 2 summarizes the proposed hormonal, tissue-level, neuromuscular, and biomechanical pathways that may contribute to non-contact ACL injury susceptibility. The figure is intended to illustrate biological plausibility rather than an established causal sequence.

Importantly, the intermediate outcomes shown in the figure—including ligament laxity, muscle-activation patterns, dynamic knee valgus, and anterior tibial translation—are mechanistic or biomechanical surrogates and are not equivalent to prospectively documented ACL injury.

In particular, current evidence does not support classifying the luteal or premenstrual phase as a consistently high-risk period for ACL injury.

The upper panel presents a generalized, illustrative hormonal profile across a typical ovulatory MC. Ovulation is shown near day 14 for illustrative purposes only because its timing and the magnitude of hormonal fluctuations vary substantially within and between individuals. Estradiol, progesterone, and relaxin may influence ligament extracellular matrix turnover, tissue mechanical properties, neural excitability, sensorimotor integration, and muscle activation strategies. Evidence for the ligament pathway is derived primarily from *in vitro*, tissue-level, preclinical, and selected human laxity studies, whereas evidence for the neuromuscular pathway remains limited and heterogeneous. During high-demand tasks such as landing, deceleration, and cutting, altered movement strategies may be associated with biomechanical surrogates of ACL loading, including dynamic knee valgus, anterior tibial translation, and anterior shear. These surrogate outcomes should not be interpreted as equivalent to prospectively documented ACL injury. Solid arrows indicate relatively established biomechanical sequences, whereas dashed arrows indicate proposed or incompletely validated hormone-related links. The figure illustrates biological plausibility and does not establish that any MC phase directly causes ACL injury or represents a consistently high-risk window. All figures were created by the author using Adobe Photoshop 2024 (version 25.0). No third-party images were used. No additional permissions are required.

### 5.2 Potential associations with other musculoskeletal injuries

Muscle strains are prevalent across sports, and within-cycle hormonal variation has been proposed as one possible modifier of muscle–tendon mechanical properties. This hypothesis is based partly on experimental evidence concerning estradiol and connective tissue metabolism, rather than on consistent prospective data showing phase-specific muscle strain incidence. One shear-wave elastography study reported greater contraction-induced muscle stiffness during menstruation than during the ovulatory window (70). However, muscle stiffness constitutes merely a biomechanical surrogate measure and cannot be directly equated with clinical muscle strain susceptibility. Prospective epidemiological data tracking muscle strain incidence across hormone-verified cycle phases remain sparse and inconsistent (61). Accordingly, no consistent phase-specific pattern of muscle strain susceptibility has been established to date, and further standardized monitoring research is required to validate this mechanistic hypothesis.

Bone stress injuries result from an imbalance between repetitive mechanical loading and the capacity of bone to remodel and repair. The most clinically relevant reproductive-hormone concern is not a transient phase within a normal ovulatory cycle, but persistent menstrual dysfunction and hypoestrogenism associated with low energy availability and relative energy deficiency in sport. These conditions may impair bone remodeling and increase susceptibility to bone stress injury (24). Prevention should therefore prioritize adequate energy availability, nutritional status, assessment of menstrual function, progressive load management, and early identification of bone-health concerns rather than phase-specific restrictions within an otherwise normal MC.

Joint laxity has also been examined across the MC, but findings are heterogeneous. One study reported greater medial and lateral knee laxity during parts of the luteal phase and associations with progesterone and relaxin concentrations (25). However, these findings have not been consistently reproduced, and a recent systematic review and meta-analysis found no overall clinically meaningful effect of the MC phase on knee laxity (4). Training status and neuromuscular adaptation may further modify the relationship between passive laxity and dynamic joint stability. Accordingly, proprioceptive and neuromuscular injury-prevention training should be maintained throughout the cycle rather than concentrated in a presumed vulnerable phase.

Overall, direct evidence linking normal within-cycle hormonal variation to the incidence of muscle strains, bone stress injuries, or other musculoskeletal injuries remains sparse. Tissue-level and biomechanical studies provide hypotheses regarding hormone-sensitive connective tissue metabolism and passive joint properties, but these findings have not established phase-specific clinical injury susceptibility. Persistent menstrual dysfunction, low energy availability, and hypoestrogenism are more clearly associated with impaired bone health; importantly, these chronic conditions are conceptually and clinically distinct from transient hormonal variation across a normal ovulatory cycle. Prospective, hormone-verified injury-surveillance studies are required before phase-specific injury claims can be made.

Representative studies examining MC-related performance, recovery, neuromuscular, and injury outcomes are summarized in Table 1. The table highlights substantial heterogeneity in study populations, phase-confirmation methods, hormonal contraceptive status, and outcome selection. Importantly, studies using calendar-based phase estimation generally provide less certain evidence than studies incorporating urinary LH testing and biochemical hormone verification.

## 6 Challenges and controversies in research methodology

### 6.1 Accuracy of cycle phase confirmation and individual variability

Accurate MC phase identification is essential for research examining exercise performance and injury susceptibility. Many previous studies relied on self-reported menstrual history or calendar counting, often assuming that ovulation occurred on a fixed day of the cycle and that luteal-phase duration was constant. These assumptions are frequently incorrect because cycle length and ovulation timing vary both between and within individuals (71). Calendar-based classification may therefore misrepresent the underlying hormonal milieu.

Substantial variability exists in cycle length, follicular- and luteal-phase duration, ovulatory status, and the amplitude of estradiol and progesterone fluctuations. Apparently, regular bleeding patterns do not independently verify ovulation or adequate luteal progesterone exposure (71). In addition, physiological responses may differ across repeated cycles within the same individual. For example, within-person variation in endothelial function across two cycles has raised concerns about concluding a single-cycle assessment (72). Multicycle repeated-measures designs are therefore preferable whenever feasible.

Current methodological recommendations support combining prospective bleeding records with urinary LH testing and biochemical measurement of estradiol and progesterone (12, 73). Wearable-derived measures, including basal body temperature, sleeping heart rate, heart rate variability, and skin or finger temperature, may provide complementary information under free-living conditions (38, 74). Computational approaches, including phase-aligned cycle time scaling and MC visualization tools, may also improve the representation of variable cycle lengths and facilitate cross-study comparisons (75, 76). However, these approaches do not remove the need for appropriate hormonal verification in mechanistic studies.

Machine-learning approaches have also been investigated to optimize the timing and interpretation of salivary hormone sampling and to improve phase-classification accuracy (77). Their performance may depend on the number and timing of samples, particularly around hormonal transitions. Nevertheless, hormone concentrations and cycle characteristics may be affected by stress, energy availability, physical activity, illness, and other contextual factors. Frequent serum sampling may be impractical in large longitudinal studies, whereas salivary assays and wearable measures are more feasible but may provide lower precision or indirect estimates (73). In addition, individual differences in hormone sensitivity and receptor expression may influence physiological responses independently of circulating concentrations (78). Future research should therefore use individualized, multimodal phase-confirmation strategies rather than relying solely on self-report or calendar counting.

### 6.2 Control of confounding factors

Research into how MC hormonal fluctuations shape exercise performance and injury susceptibility is complicated by numerous confounding variables that substantially affect these outcomes. Selected studies have reported small differences in aerobic outcomes during midluteal assessments (2, 79). However, these findings do not establish that the concurrent concentrations of estradiol and progesterone caused the observed differences; the differences may also reflect training load, nutritional status, psychological state, environmental conditions, measurement error, or normal within-person variability. Without comprehensive statistical adjustment for these confounders, observed changes in performance or injury susceptibility may be incorrectly attributed solely to cyclic hormonal shifts, hindering the translation of study results into evidence-based guidance for female athletes.

Another critical confounding factor is hormonal contraceptive use. Hormonal contraceptives alter endogenous reproductive hormone patterns, although the magnitude and nature of these changes vary according to contraceptive formulation, dose, and administration schedule. Combined oral contraceptives generally suppress ovulation and reduce the typical cyclic fluctuations in endogenous estradiol and progesterone. For example, one study reported differences in selected steady-state ventilatory responses between naturally menstruating participants and oral contraceptive users (80). Such findings may reflect formulation-specific effects, participant characteristics, or study conditions and should not be generalized to all oral contraceptive users. Survey data indicate that naturally menstruating athletes and hormonal contraceptive users may report different symptom profiles and perceived effects on exercise or performance (81). These self-reported perceptions are clinically relevant but should not be interpreted as evidence of objectively measured impairment in performance. One study identified differences in selected extracellular vesicle and metabolite responses to resistance exercise across cycling conditions and hormonal contraceptive status (82). These molecular findings are exploratory and do not demonstrate corresponding differences in training adaptation, athletic performance, or injury susceptibility. Failure to account for the distinct hormonal milieu induced by oral contraceptive use prevents researchers from drawing robust conclusions and hinders establishing evidence-based guidelines applicable to individuals with different endogenous and exogenous hormonal profiles.

## 7 Moderating role of psychological and perceptual factors

Psychological, affective, and motivational responses across the MC should not be interpreted as direct consequences of hormonal fluctuations alone. These responses are better understood through three interrelated domains: symptom perception, perceived exertion and readiness for activity, and environmental or contextual factors. This framework distinguishes the symptoms experienced by athletes from their perceived effects on exercise behavior and recognizes that these relationships are shaped by the sporting and social environment.

### 7.1 Symptom perception

Female athletes report a range of MC-related symptoms, including fatigue, dysmenorrhea, headache, irritability, negative affect, impaired concentration, and perceived cognitive dysfunction (7, 8, 42, 83, 84). These symptoms are commonly reported during the late luteal phase and during menstruation, although their timing, severity, and functional relevance vary considerably both between athletes and across cycles within the same athlete. Therefore, the MC phase alone may not reliably predict symptom burden of an individual athlete.

Changes in estradiol and progesterone have been investigated in relation to affective and perceptual variation via autonomic, thermoregulatory, and neurotransmitter pathways (16, 42, 85, 86). However, findings are heterogeneous, and circulating hormone concentrations should not be interpreted as direct or sole determinants of emotional responses. In particular, current evidence does not establish the late follicular phase as a universally more emotionally stable or psychologically favorable period. Sleep quality, training load, energy availability, previous menstrual experiences, and psychological state may also influence how symptoms are perceived and reported.

Pain is an important component of symptom perception. Selected studies have reported between-phase differences in pain sensitivity or tolerance, including higher sensitivity during some early-follicular or menstrual assessments and higher tolerance during assessments characterized by elevated estradiol and progesterone (17, 87, 88). However, the direction and magnitude of these findings vary across pain modalities, populations, and phase-confirmation methods. Psychological factors, including pain catastrophizing and expectations regarding menstrual discomfort, may further modify pain experience (89). In athletes, altered pain perception may affect symptom reporting, willingness to continue training, and decisions regarding injury management. Accordingly, pain should be assessed at the individual level rather than inferred solely from the MC phase.

### 7.2 Perceived activity, exertion, and motivational responses

MC-related symptoms may influence athletes’ perceptions of exercise, including perceived exertion and readiness to train, as well as their fatigue, motivation, and confidence. During the late luteal phase and menstruation, some athletes report increased fatigue, discomfort, or autonomic symptoms such as nausea and dizziness, which may reduce their willingness to initiate or sustain high-intensity exercise (8, 90, 91). Progesterone-related increases in basal body temperature may also contribute to greater subjective thermal strain under some exercise and environmental conditions.

Importantly, perceived impairment does not necessarily correspond to an objectively measurable reduction in athletic performance. Although some athletes report higher perceived exertion or poorer readiness during specific cycle phases, maximal strength, aerobic capacity, and other objective performance outcomes often show small, inconsistent, or no phase-related differences (8, 47, 48). The distinction between perceived activity-related effects and objectively measured performance should therefore be maintained.

Changes in symptom burden and perceived exertion may affect pacing, self-regulation, training adherence, and recovery behavior. For example, an athlete who anticipates greater fatigue may select a lower exercise intensity or terminate a session earlier, even when physiological capacity is relatively preserved. Conversely, an athlete with a higher pain tolerance may continue training despite symptoms that require clinical attention. These findings support the use of individual symptom and perceived-readiness monitoring alongside objective measures of training load and performance.

### 7.3 Environmental and contextual factors

The psychological and motivational effects associated with the MC are shaped by environmental and contextual factors. The same symptoms may have different consequences during routine training, high-stakes competition, prolonged endurance exercise, or technically demanding team-sport activities. Environmental heat, training load, sleep, recovery status, and nutritional availability may interact with menstrual symptoms, altering perceived exertion and readiness.

Social and organizational environments are also important. Female athletes frequently report discomfort discussing menstrual symptoms, particularly when communication structures are unclear or when coaching and medical teams lack sufficient knowledge of menstrual health (9, 84, 92). Menstrual stigma and fear of being perceived as weak may discourage athletes from reporting symptoms, requesting training modifications, or seeking medical support. In contrast, supportive communication, menstrual health education, and confidential monitoring systems may facilitate appropriate symptom management and individualized training decisions (9, 19, 92–94).

Expectations regarding MC-related performance may also influence symptom interpretation and activity-related decision-making. Athletes who expect a particular phase to impair performance may pay greater attention to fatigue or discomfort and may modify pacing or effort accordingly. This possibility has sometimes been described as a nocebo-like effect, but its magnitude and causal importance remain uncertain (8, 95). These expectations should therefore be addressed through balanced education that acknowledges genuine symptoms without promoting deterministic beliefs that performance must decline during a specific phase.

Hormonal contraceptive use represents an additional contextual factor. Users of combined oral contraceptives may experience mood or symptom changes during the hormone-withdrawal interval, and their symptom profiles may differ from those of naturally menstruating athletes (81, 83). Naturally, cycling athletes and hormonal contraceptive users should therefore be assessed separately, with attention to contraceptive type, formulation, and withdrawal schedule.

Overall, MC-related psychological and motivational responses emerge from interactions among perceived symptoms, activity-related perceptions, and environmental or contextual conditions. Practical management should prioritize individualized symptom monitoring, athlete-staff communication, and flexible training adjustments rather than universally reducing training according to a predetermined menstrual phase.

## 8 Practical considerations for menstrual cycle monitoring and individualized training adjustment

### 8.1 Individualized cycle monitoring and education

Individualized MC monitoring and education may support athlete health, symptom management, and informed training decisions. Cycle length, phase duration, ovulatory status, hormonal profiles, and symptom burden vary substantially both between individuals and across cycles within the same individual (12, 71). Prospective monitoring of bleeding patterns, symptoms, perceived readiness, and relevant training outcomes may help identify reproducible individual patterns. Urinary hormone monitoring and basal body temperature tracking may improve reproductive health awareness, although their accuracy and feasibility vary (27). Emerging wearable biosensors may eventually permit less invasive hormone monitoring, but their validity and practical value in athletic settings require further evaluation (96).

Education for athletes, coaches, and multidisciplinary support staff is also important for building awareness of menstrual physiology and its potential implications for training and performance. Current evidence indicates substantial knowledge gaps regarding MC phases and different hormonal contraceptive formulations among elite athletes, which may hinder informed decision-making and effective symptom management (92, 97). Preliminary evaluations suggest that menstrual health literacy programs may improve functional, interactive, and critical menstrual health literacy and are generally well received, although evidence in athletic populations remains limited (93). Digital health tools represent one form of technology-supported education. For example, a pilot randomized controlled trial of a commercially available cycle-tracking application reported improvements in menstrual health literacy and symptom recognition, although the trial was small and conducted with industry involvement (94).

Educational initiatives should also address menstrual stigma and promote open dialog to reduce barriers to communication about menstrual health in sport. Multiple qualitative studies have indicated that athletes often find it difficult to discuss menstrual concerns with male coaches and perceive a lack of formal channels for such conversations (7, 9). This situation may exacerbate feelings of isolation and impede effective symptom management (92). Normalizing conversations about menstrual health and equipping all relevant stakeholders with appropriate terminology and sufficient confidence can reduce stigma, foster a more supportive athletic atmosphere, and simplify the implementation of individualized training adjustments (9).

Individualized cycle monitoring and education may help athletes identify their own menstrual and symptom patterns and adjust training and recovery accordingly. Successful implementation requires multidisciplinary support, improved menstrual-health literacy, confidential communication pathways, and efforts to reduce stigma within the sporting environment.

### 8.2 Symptom-informed and individually responsive training adjustments

Selected studies have reported small between-phase differences in particular strength, endurance, or neuromuscular outcomes. While some of these studies found higher values during the late-follicular phase, others did not confirm this pattern (21, 50). These findings do not establish that the late-follicular hormonal profile caused the differences or that this phase is optimal for training. Other studies have reported stable jump performance, concentric velocity, perceived exertion, and strength across the MC (10, 49). Current evidence therefore does not establish the late follicular phase as a universally optimal period for high-load resistance training, plyometric exercise, or skill acquisition. Coaches may consider phase-related information only when an athlete demonstrates a reproducible individual pattern across multiple cycles. In the absence of such a pattern, training should proceed according to established periodization principles. In particular, training load should not be proactively increased during the late follicular phase solely on the assumption that elevated estradiol improves strength, endurance, mood, or motor control.

During the midluteal phase, elevated progesterone contributes to a rise in basal body temperature and may increase thermal and perceptual strain, particularly during prolonged exercise in hot environments (2). However, evidence does not consistently demonstrate reduced exercise performance or increased incidence of clinical injury during the luteal phase (10, 49, 61). Hydration, cooling, recovery time, or session density may be adjusted when an athlete repeatedly experiences heat intolerance, fatigue, sleep disturbance, or impaired recovery. A universally conservative or recovery-focused training strategy for the luteal phase is not supported. The luteal phase should likewise not be classified as a high-injury-risk window based on thermoregulatory or perceptual changes alone.

Training during menstruation should be guided primarily by symptom severity rather than bleeding status alone. In a four-season study of elite female football players, injury incidence did not differ significantly between bleeding and non-bleeding days. Injury burden was higher during bleeding days, but only 11 injuries occurred during bleeding; this finding should therefore be considered exploratory and should not be interpreted as evidence that injuries during menstruation are inherently more severe (61). Athletes with substantial pain, heavy bleeding, gastrointestinal symptoms, dizziness, or fatigue may benefit from temporary individualized adjustments and, when indicated, clinical assessment. Iron status should be evaluated when symptoms or risk factors suggest deficiency rather than based on the MC phase alone.

Overall, evidence is insufficient to recommend MC-phase-based training periodization as a routine strategy. The MC phase may be recorded as contextual information, but training decisions should be guided primarily by established periodization principles and repeated individual monitoring of symptoms, perceived exertion, recovery, training load, and objective performance. Phase-specific adjustments should be considered only when an athlete demonstrates a reproducible and functionally meaningful pattern across multiple cycles. Randomized controlled trials with accurate phase confirmation are required before universal phase-specific training recommendations can be made.

### 8.3 General nutrition principles and symptom-informed recovery

Nutrition and recovery recommendations should be based primarily on established sports nutrition principles rather than presumed menstrual-phase requirements. Across all MC phases, athletes should prioritize adequate energy availability, carbohydrate intake matched to training demands, sufficient daily protein distributed across meals, appropriate hydration, and correction of clinically identified nutrient deficiencies (13, 14, 30, 31). These are general sports nutrition recommendations and should not be interpreted as interventions shown to improve performance or recovery when prescribed according to a particular MC phase.

Changes in resting energy expenditure, appetite, substrate metabolism, body temperature, or amino-acid metabolism across the MC provide biologically plausible reasons to investigate phase-related nutritional requirements. However, direct intervention evidence showing that routine phase-specific increases in total energy, carbohydrate, protein, antioxidant intake, or supplementation improve athletic performance or recovery remains limited. Accordingly, athletes should not automatically increase energy or macronutrient intake during the luteal phase solely based on expected hormonal changes. Individual adjustments may be considered when repeated monitoring identifies greater hunger, reduced dietary intake, heat intolerance, gastrointestinal symptoms, fatigue, or impaired recovery, but these adjustments should also account for training load, energy availability, environmental conditions, and habitual dietary intake.

Recovery support should similarly be guided by the athlete's symptoms and recovery data rather than the MC phase alone. Sleep opportunity, postexercise nutrition, hydration, cooling, and session spacing may be adjusted when an athlete demonstrates reproducible difficulties, but current evidence does not support a universal phase-specific recovery protocol (15, 17, 47, 48). Iron assessment should be based on menstrual blood loss, dietary intake, symptoms, and biochemical markers, and iron supplementation should not be prescribed solely because an athlete menstruates (28–31). One small crossover trial involving 15 CrossFit® athletes reported differences in selected performance, cognitive, and soreness outcomes after short-term dark chocolate supplementation (98). Because of the small sample size, incomplete hormonal verification, and lack of independent replication, these findings should be regarded as exploratory and do not support routine or phase-specific dark chocolate supplementation.

Overall, the evidence is insufficient to prescribe phase-specific nutritional or recovery strategies. General sports nutrition principles should be maintained throughout the cycle, with modifications made only when repeated individual monitoring identifies clinically or practically meaningful symptoms, dietary difficulties, or recovery problems. Phase-related symptom considerations and the corresponding practical recommendations are summarized in Table 3.

## 9 Future research directions and clinical application prospects

### 9.1 Deepening mechanistic research and establishing personalized predictive models

Future mechanistic research should use longitudinal, repeated-measures designs that combine accurate hormonal assessment with relevant physiological, biomechanical, and performance outcomes. Inaccurate phase classification, small sample sizes, single-cycle testing, and inadequate control of confounding variables have contributed substantially to inconsistent findings (1, 12). Multiomics approaches, including metabolomics and extracellular-vesicle profiling, may help characterize systemic responses to exercise across different hormonal environments (82). However, such molecular markers should be linked to clinically meaningful outcomes rather than interpreted in isolation.

Heterogeneous individual responses across MC phases provide a rationale for investigating personalized predictive models. Future research should determine whether such models can predict clinically meaningful changes in symptoms, readiness, performance, or injury outcomes more accurately than established monitoring approaches. For example, future studies could evaluate whether combining hormone-related measures with biomechanical, physiological, symptom, and workload data improves individualized training or rehabilitation decisions. At present, wearable hormone-monitoring technology remains in development, and evidence that it improves performance or reduces injury risk is lacking (96). Genetic markers related to hormone signaling or collagen metabolism could also be investigated as candidate predictors. However, current evidence does not demonstrate that their inclusion improves the prediction of ligament injury beyond established clinical, biomechanical, exposure, and previous-injury variables.

Personalized models should be developed and validated using prospective cohorts followed across multiple cycles. Phase-classification protocols should combine prospective bleeding records, urinary LH testing, and, where feasible, estradiol and progesterone measurements (12). Diverse populations should be included across sports, training levels, age groups, menstrual characteristics, and hormonal contraceptive status. Models should also undergo external validation and calibration before being used to guide training or clinical decisions. Continuously updated approaches may eventually support adaptive interventions, but athlete privacy, data governance, interpretability, and risks of algorithmic misclassification must also be addressed.

Mechanistic evidence may inform feature selection for predictive models, but variables should be retained only when they demonstrate reproducible associations with meaningful outcomes. For instance, selected studies have examined associations between estradiol concentrations and strength outcomes or between luteal-phase hormonal profiles and perceived exertion or substrate metabolism (33, 50). Other studies have assessed inflammatory or muscle-damage biomarkers across the MC (11). However, these associations are inconsistent, and none of these biomarkers has been validated as a standalone indicator for adjusting training load or preventing injury.

Hormone concentrations, inflammatory markers, muscle-damage biomarkers, and biomechanical measures should not automatically be treated as proxies for performance impairment or injury. Subjective symptoms, including pain sensitivity and mood changes, may complement objective monitoring data and improve model performance (79). Prospective validation is required to determine whether such models improve decisions beyond established workload monitoring and clinical assessment.

Rigorous mechanistic research and appropriately validated predictive models may help determine whether hormone-related information adds value to established symptom, workload, and clinical monitoring. Their ability to improve training decisions, performance, or injury prevention has not yet been established. Large longitudinal datasets and repeated hormone-verified assessments will be essential for developing clinically useful tools.

### 9.2 Validation of cycle-informed interventions

Evidence supporting menstrual-phase-specific training, nutrition, supplementation, and recovery interventions remains limited. Most proposed protocols are based on mechanistic reasoning, small observational studies, or preliminary interventions rather than adequately powered randomized controlled trials. Future trials should compare fixed phase-based approaches, individualized symptom-informed adjustments, and conventional practice, with accurate phase verification, standardized interventions, predefined clinically meaningful outcomes, and appropriate control of training load, energy availability, sleep, and hormonal contraceptive status.

Nutritional trials should distinguish between interventions intended to correct general dietary inadequacy and those interventions specifically designed to modify phase-related symptoms or performance. Similarly, neuromuscular and rehabilitation studies should compare phase-specific or symptom-informed modifications with established year-round injury-prevention programs rather than assume that a particular phase requires additional treatment. Until replicated trials demonstrate clear benefits, universal phase-specific training, nutritional supplementation, or recovery protocols cannot be recommended.

### 9.3 Expanding study populations and application scope

Existing studies frequently involve small samples of young, healthy, eumenorrheic participants, many of whom are recreationally active rather than elite athletes. This limits generalizability across different sports, competition levels, age groups, menstrual characteristics, and hormonal contraceptive users. Future studies should include adolescent athletes, elite and recreational athletes, athletes with menstrual dysfunction, and users of different hormonal contraceptive formulations, while analyzing these groups separately where appropriate.

Menstrual health should be incorporated into long-term athlete health management and career planning to advance more inclusive, individualized practice within sports science. Menstrual regularity, bleeding characteristics, and related symptoms may provide useful information about reproductive and general health and may help identify menstrual dysfunction, low energy availability, or other concerns requiring further assessment. However, these variables should not be treated as direct measures of competitive readiness, athletic performance, or injury susceptibility. Importantly, the MC phase ought not to be used as an independent indicator of training or competition preparedness. Comprehensive monitoring frameworks need to combine menstrual data with training load, performance metrics, recovery status, nutritional intake, sleep quality, and clinical records. Such an integrated framework may support more comprehensive and individualized athlete-health management, but its effects on career longevity, performance stability, and injury incidence require prospective evaluation.

A recent systematic review and meta-analysis found no significant overall effect of exercise on free estradiol or serum progesterone in healthy reproductive-age women, although testosterone responses were observed (99). Future intervention studies should use standardized exercise protocols, appropriate comparison conditions, accurate characterization of menstrual and hormonal contraceptive status, and, where feasible, blinded outcome assessment. These methodological improvements may help clarify how acute and chronic exercise interact with reproductive-hormone profiles.

## 10 Conclusion

This narrative review indicates that MC-related fluctuations in estradiol and progesterone have been investigated in relation to metabolism, thermoregulation, neuromuscular function, connective-tissue properties, pain, mood, and perceived exertion. However, their associations with objectively measured athletic performance, recovery, and clinical musculoskeletal injury outcomes are generally weak, inconsistent, task-dependent, and highly individual. Current evidence does not identify any MC phase as universally advantageous or disadvantageous for performance, or consistently associated with increased susceptibility to clinical injury, including ACL injury. Mechanistic findings and laboratory surrogates, such as hormone concentrations, ligament laxity, cortical excitability, muscle activation, and landing biomechanics, should not be treated as evidence of phase-specific performance impairment or clinical injury risk unless they are prospectively linked to those outcomes. Therefore, rigid phase-based prescriptions for training, nutrition, recovery, or injury prevention are not supported. MC information should instead be treated as a component of a broader individualized monitoring framework that includes symptoms, readiness, objective performance, workload, energy availability, sleep, environmental stressors, and previous injury. Future studies should use adequately powered, prospective, multicycle designs with biochemical phase verification, distinguish naturally menstruating athletes from users of different hormonal-contraceptive formulations, and control for major confounding factors. At present, individualized, symptom-informed management is more defensible than universal menstrual-phase-specific recommendations, although the effectiveness of such individualized approaches also requires prospective evaluation.
